# Do Electronic Health Literacy and Online Health Information-Seeking Behavior Mediate the Effects of Socio-Demographic Factors on COVID-19- and Non-communicable Disease-Related Behaviors Among Myanmar Migrants in Southern Thailand?

**DOI:** 10.7759/cureus.49090

**Published:** 2023-11-20

**Authors:** Hein Htet, Wit Wichaidit, Hutcha Sriplung, Kyaw Ko Ko Htet, Aungkana Chuaychai, Tida Sottiyotin, Virasakdi Chongsuvivatwong

**Affiliations:** 1 Department of Epidemiology, Prince of Songkla University, Hat Yai, THA; 2 Department of Pharmaceutical Care, Walailak University, Nakhon Si Thammarat, THA

**Keywords:** myanmar migrants, non-communicable disease, covid-19, socio-demographic factors, online health information-seeking, electronic health literacy

## Abstract

Introduction

Myanmar migrants in Thailand are vulnerable to COVID-19 and non-communicable disease (NCD) risk behaviors, influenced by socio-demographic factors. In the digital age, migrants can seek extensive health information online, and their ability to understand and use electronic health information, which is known as electronic health literacy (e-Health literacy), becomes critical in making decisions about their health behaviors. This study aims to investigate the potential mediating roles of online health information-seeking and e-Health literacy in the associations between socio-demographic factors and COVID-19- and NCD-related behaviors.

Methods

Our study was conducted in 2022, involving 1,050 Myanmar migrants in two southern Thai cities. Data on socio-demographic factors, e-Health literacy, online health information seeking, COVID-19-related behaviors (adherence to COVID-19 protective behavior (CPB), vaccination), and NCD risk behaviors (smoking, betel chewing, alcohol consumption, substance abuse) were collected. Structural equation modeling (SEM) was employed to analyze the hypothesized relationships.

Results

Nearly all migrants received the COVID-19 vaccination in two doses and above, with reasonable good adherence to CPB. Migrants exhibited risky NCD-related behaviors, including current smoking (26.8%), alcohol consumption (17.5%), and betel chewing (25.8%). Approximately three-quarters (73.4%) had a limited e-Health literacy level, and the vast majority did not search for online health information. Their COVID-19- and NCD-related behaviors were directly influenced by socio-demographic factors without the significant mediation roles of e-Health literacy and online health information seeking.

Conclusions

Myanmar migrant workers in Southern Thailand had reasonably good practices in COVID-19-related behaviors despite engaging in risky NCD-related behaviors. These outcome behaviors were directly influenced by their socio-demographic factors, without the significant mediation roles of e-Health literacy and online health information seeking. The findings suggest that diverse interventions beyond e-Health strategies for future pandemic mitigation and enhancement of their health behaviors are needed.

## Introduction

Thailand is a top destination for international migrants in Southeast Asia [1]. Nationals from Myanmar constitute 69% of legal migrants in Thailand [2], mostly working in low-skilled jobs such as construction, factories, and fisheries [2]. During different COVID-19 waves in Thailand, Myanmar migrants were mostly impacted in terms of the number of COVID-19 infections from the wider migrant community in Thailand [3].

Generally, migrants' health problems and health behaviors are predominantly influenced by social determinants of health or socio-demographic factors, including education, occupation, income, housing, and working environment [1]. For instance, during the COVID-19 pandemic, migrants in Thailand were faced with certain challenges in practicing and adhering to physical distancing due to the unsanitary, crowded living conditions [4], as well as low COVID-19 vaccination rates, which were attributed to language and financial barriers [5]. Additionally, stressful working and poor living conditions make them vulnerable to non-communicable diseases (NCDs) and associated risk behaviors, such as tobacco and harmful alcohol use [6], which are highly prevalent not only in the Thai population [7] but also among Myanmar migrants [8].

Health literacy, one such important social determinant, is also a key contributor to migrants' health behaviors [1] and was found to be limited among Myanmar migrants in Thailand [9]. In the digital era, migrants can search for extensive health information via relevant social media, websites, and mobile applications. Therefore, moving beyond traditional health literacy, the capacity to find, understand, and use electronic health information and services, which is known as electronic health literacy (e-Health literacy) [10], becomes critical in influencing and mediating COVID-19- and NCD-related behaviors [11-14].

However, the role of e-Health literacy and online health information seeking in COVID-19- and NCD-related behaviors remains relatively unexplored, especially among vulnerable Myanmar migrants in Southern Thailand. Therefore, this study aims to investigate the potential mediating roles of e-Health literacy and online health information seeking and the associations between socio-demographic factors and COVID-19- and NCD-related behaviors. Understanding these mediating roles would help the stakeholders in designing tailored interventions and policies to be distributed through online platforms.

## Materials and methods

Study setting and design

This survey was conducted in Hat Yai and Pattani cities in Southern Thailand from September 2022 to January 2023. According to the 2021 Thailand Employment Statistics, there were 19,810 and 4,122 legal Myanmar migrant workers granted by cabinet resolution in Songkhla and Pattani provinces, respectively [15]. During the COVID-19 pandemic (2020-2022), industries in Thailand were transiently affected, and the government issued several control measures, including social distancing, compulsory mask-wearing, and workplace disinfection, to prevent further spread among workers. In late 2021, the government offered vaccinations to migrant workers free of charge as part of an inclusive vaccine policy [16]. When the COVID-19 outbreaks subsided, we conducted a cross-sectional survey among Myanmar migrants who were currently living and working in factories and construction sites in Hat Yai and fishery sites in Pattani. Other types of migrant workers, such as those in agriculture, forestry, hotels, food vendors, domestic work, and entertainment, were too scattered to collect the data. The inclusion criteria for study participants were being 18 years of age or older, having Myanmar nationality, either documented or undocumented, residing and working in study areas for at least six months, and being able to communicate in the Burmese language. Those who were already arrested for illegal reasons, those with mental or hearing problems, and those who had already been vaccinated for COVID-19 infection outside of Thailand were excluded from the study.

Sample size determination and sampling procedure

The sample size was estimated using the single population proportion formula with the “Epicalc” package in R software version 4.0.2 (The R Foundation, Vienna, Austria). The cluster sampling method was applied to accommodate the large size and wide dispersion of the migrant population. Assuming a 50% proportion of migrants with sufficient e-Health literacy level (since there was no previous research conducted in this study population in Thailand), a confidence level of 95%, a type I error of 5%, a margin of error of 5%, a non-response rate of 20%, and a design effect of 2, a minimum sample size of 922 migrant workers was required. Ultimately, a total of 1,050 migrant workers participated in this study. The sampling process began by constructing a sampling frame for the Hat Yai factory and construction worker communities and the Pattani fishery worker community, based on information obtained from the Department of Industrial Works (Thailand) website and local key informants. Subsequently, the investigators contacted these sites and sent official request letters. The stratified cluster sampling method was then applied with a sampling fraction of 0.6 for factory workers (600 out of 1,000), 0.3 for construction workers (300 out of 1,000), and 0.1 for fishery workers (100 out of 1,000) for the reasons of feasibility and time constraints during the COVID-19 pandemic. Finally, a two-stage probability-proportional-to-size cluster sampling was applied for each stratum.

Study instrument

A self-administered structured paper-based questionnaire in Burmese (30-40 minutes) was used to collect data about socio-demographic characteristics (10 items), e-Health literacy (eight items), online health information-seeking behavior (six items), NCD risk behaviors (four items), self-reported COVID-19 protective behavior (CPB, 17 items), and COVID-19 vaccination (two items). The questionnaire was developed based on guidelines from the World Health Organization (WHO) [17-19], the Center for Disease Control (CDC) [20], and the Ministry of Public Health, Thailand (MOPH) [21,22], as well as previously published literature. The content validity of the questionnaires was examined by public health experts and senior epidemiologists, and modifications were made accordingly based on their recommendations. The questionnaire was originally developed in English, forward-translated into Burmese, and then back-translated into English to check the linguistic validity of the translation. The two English versions were compared to clarify and identify the differences in all items. The reliability and understanding of items in the questionnaire were assessed through pilot testing among a snowball sample of 30 Myanmar migrant workers in Hat Yai City who were not included in the main study. Then, the items and wording of the questionnaires were modified accordingly.

Independent variables

These were socio-demographic characteristics of the participants, which were observed variables, including age, gender, ethnicity, religion, education, marital status, occupation, monthly personal income, living status, and duration of living in Thailand.

Intermediary variables

E-Health Literacy

This latent intermediary variable was measured using the previously validated eight-item e-HEALS scale [23], with participants rating items on a 5-point Likert scale (from “strongly disagree” to “strongly agree”) with a score ranging from 1 to 5. Total scores ranged from 8 to 40 and were dichotomized into "limited" (scores 8 to 26) and "sufficient" (scores 27 to 40) levels. The Cronbach’s alpha coefficient for this scale was 0.95, indicating high internal consistency. We used the total e-Health literacy score for subsequent analysis, validating it through confirmatory factor analysis (CFA).

Online Health Information-Seeking Behavior

It was also regarded as a latent intermediary variable, comprising observed binary (yes/no) items related to searching for health information through various channels such as websites, Facebook or YouTube, Viber or Line apps, SMS subscriptions, call centers, and mobile health apps. For further analysis, this latent variable was identified via exploratory factor analysis (EFA) and validated by CFA.

Outcome variables

Adherence to CPB

This was one of the three primary outcomes of the study. It included eight items measuring CPB at the workplace (wearing face masks, sanitizing hands and personal items, covering the face during coughing or sneezing, and physical distancing during working and eating at the workplace) and nine items at the residence (wearing face masks when leaving the house, opening windows to improve ventilation, sanitizing hands and frequently touched objects, covering the face during coughing or sneezing, washing clothes upon returning from work, avoiding going out of home unnecessarily, avoiding crowded places, and traveling outside of the city) over the past seven days prior to the survey. The adherence question items were adapted and modified from “survey tool and guidance: rapid, simple, flexible behavioral insights on COVID-19” by the WHO [24], the Indian study that developed and validated a tool to assess COVID-19 preventive practices [25], as well as from sources such as the WHO [17-19], CDC [20], and MOPH, Thailand [21,22]. The question items were self-reported in nature, with five answer options (rarely = less than 10% times, occasionally = 25% times, commonly = 50% times, mostly = 75% times, always = more than 90% times). Each adherence item was scored 1 for the responses “mostly and always" and zero otherwise and then summed up to create a composite adherence score for workplace and residence separately. Reliability scores (Cronbach’s alpha) of workplace and residence adherence to CPB were 0.9 and 0.7, respectively, indicating good internal consistency. CPB adherence was then treated as a latent variable for further analysis and validated by CFA.

COVID-19 Vaccination

This observed outcome variable was self-reported as the doses of COVID-19 vaccine received by the participants and assessed by the question, “How many doses of COVID-19 vaccines have you received?” with the answer options of one to four doses.

NCD Risk Behavior

This latent outcome variable was constructed using four observed variables: alcohol drinking, smoking, betel chewing, and substance abuse. Participants were first asked whether they engaged in these risk behaviors, with the answer options “never,” “previously practiced (those who reported practicing at least once in their lifetime but not in the past 30 days),” and “currently practicing” (those who reported practicing in the past 30 days).” Then, for further analysis, a latent NCD risk behavior variable underlying these four observed variables was identified via EFA and validated by CFA.

Statistical analysis

Proposed Model of Serial Mediation Analysis

**Figure 1:**
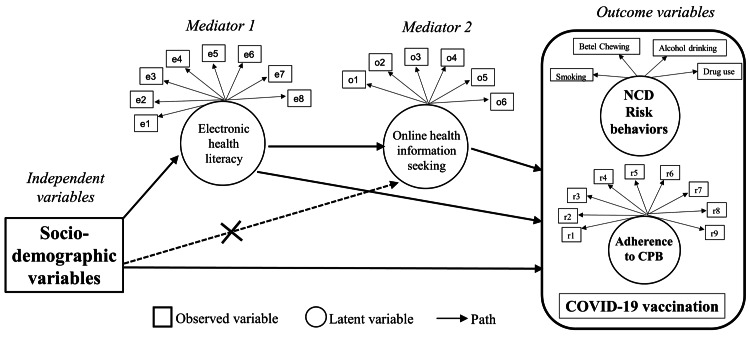
The hypothesized model framework concerning the serial mediation effects of e-Health literacy and online health information seeking on the associations between socio-demographic characteristics and COVID-19- and NCD-related outcomes NCD: non-communicable disease

A serial mediation model framework was proposed (Figure 1), suggesting migrants’ socio-demographic characteristics could impact COVID-19- and NCD-related outcomes, directly or indirectly, through two latent intermediary variables: e-Health literacy and online health information seeking. We hypothesized that serial mediation was present if the indirect effects in the pathway from socio-demographic factors → e-Health literacy → online health information seeking → COVID-19- and NCD-related behaviors were statistically significant, excluding the pathway from socio-demographic factors → online health information seeking → COVID-19- and NCD-related behaviors.

Data entry and analysis

EpiData software version 3.1 (EpiData Association, Denmark) and R software version 4.0.2 were used for data entry and analysis. All independent, intermediary, and outcome variables were first examined for their distribution to gain insights into the general information. The proposed model of the serial mediation framework, as shown in Figure 1, was analyzed using the structural equation modeling (SEM) technique, which involved the following two-step approach.

Step 1: Assessment of the Measurement Model

The first step of the SEM technique was the measurement model, which established the relationship between observed and latent variables using both EFA and CFA. We randomly split the dataset in half and performed EFA first to extract the new latent construct “NCD risk behavior” using four observed variables, as well as another new latent construct “online health information seeking” using six observed variables. EFA was performed by the “psych” R package using the maximum likelihood factoring method [26] with a varimax rotation approach. Preliminary to EFA, the Kaiser-Meyer-Olkin (KMO) measure of sampling adequacy (cut-off above 0.6) and the Bartlett test of sphericity (significant level of p<0.05) were checked to determine whether EFA was applicable or not [27]. Then we determined the number of factors to retain by applying multiple factor extraction rules, including Kaiser's criteria for eigenvalues larger than one, a scree plot, parallel analysis (PA), and Velicer’s minimum average partial (MAP) test [27]. We removed items with factor loadings of less than 0.30 [28] and those with cross-loadings of 0.32 or greater on two or more factors [29]. Then, we tested the reliability of items in the extracted factor using Cronbach’s alpha reliability coefficient. After applying EFA, CFA was conducted on the remaining half of the dataset to test the fitness of observed items, the reliability and validity of the newly constructed latent variables (NCD risk behavior, online health information-seeking behavior), and the previously developed two latent variables (e-Health literacy, adherence to CPB). The “Lavaan” and “SemTools” packages were used for conducting CFA in R software. The validity and fitness of the measurement model were assessed with the standard criteria guided by Hu and Bentler [30] with the following model fit indices: standardized root mean square residual (SRMR close to 0.08 or below), root mean square error of approximation (RMSEA close to 0.06 or below), and comparative fit index (CFI) and Tucker-Lewis index (TLI) values (close to 0.95 or greater).

Step 2: Hypothesis Testing With the Structural Model (Structural Path Analysis)

After evaluating the measurement model, we specified the structural model to test the potential direct, indirect, and total effects in the proposed serial mediation model framework simultaneously. With the "Lavaan" and "SemTools" R packages, the SEM approach with the weighted least squares mean and variance-adjusted estimation was conducted to accommodate the use of some categorical and ordinal variables. The standardized path coefficients “β” were interpreted in terms of SD differences and presented in the path diagram. The direct, indirect, and total effects were classified into three groups based on the magnitudes of standardized path coefficients: weak (approximately 0.10), moderate (around 0.30), and strong (>0.50) [31]. The same model fit indices used in the measurement model were also applied to evaluate the fitness of the structural model. A p-value of <0.05 was considered statistically significant. The “lavaanPlot” R package was used for the visualization of the path diagram.

Ethical consideration

This study was conducted in line with the principles of the Declaration of Helsinki to protect the human rights of the participants, following ethical approval by the Institutional Ethics Committee of the Faculty of Medicine, Prince of Songkla University, Hat Yai, Thailand (REC. 65-071-18-1). Participants' information sheets were provided in Burmese, and only verbal consent was obtained due to the sensitivity and confidentiality of their immigration status. Investigators assured participants that their decision to participate would not affect their immigration status, and they could withdraw at any time. Anonymity and confidentiality were rigorously maintained, and all participants received disposable face masks and alcohol hand gels for joining the study.

## Results

Independent variables (socio-demographic characteristics)

Table 1 summarizes the background characteristics of 1,050 participants. Their mean age was 33.84 (SD = 8.24) years. The majority of the migrants were males (56.48%), Burmese (53.8%), Buddhists (95.9%), married (59.9%), secondary school level (34.7%), and factory workers (63.7%). The median monthly personal income was 9500 Baht, with an interquartile range (IQR) of 8,000 to 10,000 Baht. The majority of participants lived with family and relatives (61.2%) and resided in Thailand for less than five years (53.2%).

**Table 1:** Independent variables (socio-demographic characteristics of the respondents) (n = 1,050) ^a^Others included Chin, Kayan, Palaung, Pao, Shan ethnicity IQR: interquartile range

Variable	Description	n (%)
Age (years)		
	18-30	411 (39.14)
	31-43	508 (48.38)
	44-59	131 (12.48)
	Mean ± SD (range)	33.84 ± 8.24 (18-59)
Sex		
	Male	593 (56.48)
	Female	457 (43.52)
Ethnicity		
	Burmese	565 (53.8)
	Rakhine	206 (19.6)
	Mon	107 (10.2)
	Kayin	86 (8.2)
	Htawei	48 (4.6)
	Others^a^	10 (1)
	Missing	28 (2.7)
Religion		
	Buddhist	1,007 (95.9)
	Christian	21 (2)
	Hindu	4 (0.4)
	Islam	2 (0.2)
	Missing	16 (1.5)
Marital status		
	Married	629 (59.9)
	Single	377 (35.9)
	Divorced	15 (1.4)
	Widowed	8 (0.8)
	Separated	3 (0.3)
	Missing	18 (1.7)
Education		
	Illiterate	57 (5.4)
	Can read and write	178 (17)
	Primary	157 (15)
	Secondary	364 (34.7)
	High school	207 (19.7)
	University	48 (4.6)
	Missing	39 (3.7)
Occupation		
	Construction	243 (23.1)
	Factory	669 (63.7)
	Seafarers	137 (13.1)
	Missing	1 (0.1)
Monthly personal income (Baht)		
	<8000	328 (31.2)
	8001-10000	537 (51.1)
	>10000	185 (17.6)
	Median (IQR)	9,500 (8,000-10,000)
Living status		
	Alone	199 (19)
	With family and relatives	643 (61.2)
	With friends	207 (19.7)
	Missing	1 (0.1)
Duration of living		
	Less than 5 years	558 (53.2)
	5 years and above	492 (46.8)

Intermediary variables (e-Health literacy and online health information-seeking behavior)

As shown in Table 2, the mean e-Health literacy score was 20.83 (SD = 7.16) out of 40 (range: 8 to 40). Nearly three-quarters of the participants disagreed with all eight eHEALS items. Average scores were low for all items (mean score range: 2.57 to 2.63), particularly for the two items related to knowledge of online health resources and confidence in using online information. Approximately three-quarters (73.4%) had limited e-Health literacy (e-HEALS score <27). The vast majority did not search for online health information, especially through SMS subscriptions (99.0%) and mobile health apps (99.4%).

**Table 2:** Intermediary variables (e-Health literacy, online health information-seeking behavior among participants) (n = 1,050) e-Health: electronic health

Variable	n (%)					Mean ± standard deviation
e-Health literacy item	Strongly disagree	Disagree	Neither	Agree	Strongly agree	
1. I know how to find helpful health resources online	117 (11.1)	509 (48.5)	106 (10.1)	310 (29.5)	8 (0.8)	2.60 ± 1.05
2. I feel confident in using information from online to make health decision	117 (11.1)	528 (50.3)	94 (9.0)	297 (28.3)	14 (1.3)	2.58 ± 1.06
3. I know where to find helpful health resources online	106 (10.1)	516 (49.1)	125 (11.9)	294 (28.0)	9 (0.9)	2.60 ± 1.03
4. I know how to use online to answer my health questions	109 (10.4)	515 (49.0)	102 (9.7)	308 (29.3)	16 (1.5)	2.63 ± 1.06
5. I know what health resources are available online	102 (9.7)	535 (51.0)	131 (12.5)	273 (26.0)	9 (0.9)	2.57 ± 1.01
6. I can tell high-quality from low-quality health resources online	97 (9.2)	450 (42.9)	279 (26.6)	211 (20.1)	13 (1.2)	2.61 ± 0.95
7. I know how to use the health information I find online to help me	102 (9.7)	469 (44.7)	214 (20.4)	246 (23.4)	19 (1.8)	2.63 ±1.00
8. I have the skills I need to evaluate the health resources I find online	101 (9.6)	473 (45.0)	226 (21.5)	238 (22.7)	12 (1.1)	2.61 ± 0.98
Online health information seeking item	No	Yes				
1. Searching health information via websites	684 (65.1)	366 (34.9)				
2. Searching health information via Facebook/YouTube	480 (45.7)	570 (54.3)				
3. Searching health information via Viber/Line	1014 (96.6)	36 (3.4)				
4. Searching health information via SMS subscription	1040 (99.0)	10 (1.0)				
5. Searching health information via call center	1039 (99.0)	11 (1)				
6. Searching health information via mobile health app	1044 (99.4)	6 (0.6)				

Outcome variables (COVID-19- and NCD-related behaviors)

Table 3 shows that mean scores (±SD) for adherence to CPB were 6.7 ± 2.0 at residence and 4.6 ± 3.1 at the workplace. Almost all migrants (97.2%) had received two or more doses of the COVID-19 vaccine. The proportions of current smokers, alcohol consumers, betel chewers, and illicit drug users were 26.8%, 17.5%, 25.8%, and 0.7%, respectively.

**Table 3:** Outcomes variables (COVID-19- and NCD-related behaviors among participants) (n = 1,050) NCD: non-communicable disease, CPB: COVID-19 protective behavior

Variable	Description	n (%)
NCD-related behaviors		
Smoking		
	Never	712 (67.8)
	Previous	57 (5.4)
	Current	281 (26.8)
Alcohol drinking		
	Never	756 (72)
	Previous	110 (10.5)
	Current	184 (17.5)
Betel chewing		
	Never	696 (66.3)
	Previous	82 (7.8)
	Current	272 (25.9)
Use of illicit drug		
	Never	1027 (97.8)
	Previous	16 (1.5)
	Current	7 (0.7)
COVID-19-related behaviors		
Adherence to CPB scores at residence, mean ± standard deviation (range)		6.7 ± 2.0 (0 - 9)
Adherence to CPB scores at workplace, mean ± standard deviation (range)		4.6 ± 3.1 (0 - 8)
COVID-19 vaccination		
	1 dose	29 (2.8)
	2 doses	456 (43.4)
	3 doses	329 (31.3)
	4 doses	236 (22.5)

SEM analysis for testing serial mediation effects

The Measurement Model

Table 4 shows the results of EFA first. The KMO value was 0.69, and Bartlett’s test was significant (χ2 = 333.87, df = 6, p<0.0001), revealing EFA was applicable to NCD risk behavior. The eigenvalues, the scree plot, PA, and MAP criteria were in agreement that one factor was sufficiently retained. The emerging factor contained three items (smoking, alcohol drinking, and betel chewing), with factor loadings ranging from 0.631 to 0.749. The communality values were higher than 0.3, indicating three variables were well represented by the extracted factors [29,32]. This one-factor model explained 36.2% of the total variance. Cronbach’s alpha reliability coefficient, which was 0.72, showed acceptable internal consistency. We simply named the extracted factor "NCD risk behavior."

**Table 4:** EFA of latent variables NCD: non-communicable disease

Items		Factor loading	Communality	Cronbach’s alpha	Proportion of total variance
NCD risk behavior				0.720	0.362
	Smoking	0.665	0.442		
	Betel chewing	0.631	0.398		
	Alcohol drinking	0.749	0.560		
	Use of illicit drug	0.221	0.047		
Online health information-seeking behavior				0.720	0.200
	Searching via websites	0.823	0.678		
	Searching via Facebook/YouTube	0.681	0.463		
	Searching via Viber/Line	0.218	0.047		
	Searching via SMS subscription	0.083	0.006		
	Searching via call center	0.083	0.006		
	Searching via mobile health app	0.104	0.010		

For the EFA of online health information seeking, the KMO value was 0.54, which was below the cut-off value; however, Bartlett’s test was significant (χ2 = 260.46, df = 15, p<0.0001). We only retained one factor, based on the MAP criterion, and the emerging factor contained two items (searching via websites and Facebook/YouTube), with factor loadings ranging from 0.681 to 0.823. The communality values were higher than 0.3. This one-factor model explained 20% of the total variance. Cronbach’s alpha reliability coefficient was 0.72. We simply named the extracted factor “online health information-seeking behavior.”

As shown in Table 5, CFA Model 1 revealed that all items had factor loadings over 0.3, except for items 7, 8, and 9 of adherence to CPB at the residence scale. Initially, this model had poor fit indices (χ2 = 2486.616, df = 368, p<0.001, CFI = 0.821, TLI = 0.802, RMSEA = 0.105, SRMR = 0.067). After evaluating model fit, internal consistency, reliability, and discriminant validity were determined. Cronbach’s alpha reliability coefficients for all constructs were above 0.7 (range: 0.753-0.958). The values of average variance extracted (AVE) as a convergent validity test were greater than 0.5, except for adherence to CPB at the residence scale. Therefore, we modified CFA Model 1 by deleting those three items (items 7, 8, and 9) of adherence to CPB at the residence scale, and CFA Model 2 was run. Factor loadings of all items in Model 2 were greater than 0.3. Model 2 demonstrated acceptable fit indices (χ2 = 290, df = 290, p<0.001, CFI = 0.868, TLI = 0.852, RMSEA = 0.098, SRMR = 0.044), though RMSEA was a little bit larger than the cut-off value of 0.06. Cronbach’s alpha reliability coefficients were greater than 0.7, and all AVE values were higher than 0.5, revealing the observed variables in Model 2 effectively measured the intended latent constructs.

**Table 5:** CFA of latent variables CFA: confirmatory factor analysis, NCD: non-communicable disease, CPB: COVID-19 protective behavior, CFI: comparative fit index, TLI: Tucker-Lewis index, RMSEA: root mean square error of approximation, SRMR: standardized root mean square residual, N/A: not applicable

Construct and items		CFA model 1		CFA model 2	
		Factor loading	Cronbach’s alpha	Factor loading	Cronbach’s alpha
Online health information-seeking behavior			0.739		0.739
	Searching via websites	0.698		0.701	
	Searching via Facebook/YouTube	0.841		0.838	
e-Health literacy			0.958		0.959
	I know how to find helpful health resources online	0.897		0.897	
	I feel confident in using information from online to make health decision	0.898		0.898	
	I know where to find helpful health resources online	0.908		0.908	
	I know how to use online to answer my health questions	0.923		0.923	
	I know what health resources are available online	0.882		0.882	
	I can tell high quality from low-quality health resources online	0.815		0.815	
	I know how to use the health information I find online to help me	0.777		0.777	
	I have the skills I need to evaluate the health resources I find online	0.765		0.765	
NCD risk behavior			0.753		0.754
	Smoking	0.641		0.641	
	Betel chewing	0.714		0.713	
	Alcohol drinking	0.791		0.791	
Adherence to CPB at residence			0.825		0.852
	Wearing face masks while going out of home	0.698		0.700	
	Regular opening of windows in your home to improve airflow	0.528		0.532	
	Sanitizing hands with soap and water/ alcohol-based sanitizer after touching eyes/nose/mouth	0.804		0.809	
	Covering face with a handkerchief/ bent elbow while coughing/sneezing	0.828		0.825	
	Sanitizing frequently touched objects and surfaces in your home	0.808		0.802	
	Washing clothes worn in workplace when back from work	0.508		0.513	
	Avoid going out of the house unnecessarily	0.318		N/A	
	Going to crowded places such as markets, shopping malls	0.276		N/A	
	Traveling outside your city within a week	0.192		N/A	
Adherence to CPB at workplace			0.941		0.942
	Wearing a face mask in your workplace	0.734		0.734	
	Cleaning hands before or after touching eyes/nose/mouth	0.805		0.805	
	Sanitizing hands with soap and water/ alcohol-based sanitizer	0.835		0.834	
	Washing hands for at least 20 seconds	0.828		0.828	
	Covering face with a handkerchief/ bent elbow while coughing/sneezing	0.782		0.781	
	Sanitizing personal items (e.g., your table, your instruments) used in your workplace	0.858		0.848	
	Maintaining a minimum distance of 1 meter at workplace	0.847		0.848	
	Maintaining a minimum distance of 1 meter while eating food with your colleagues at your workplace	0.851		0.851	
CFA Model 1 Fit indices: CFI = 0.825, TLI = 0.807, RMSEA = 0.098, SRMR = 0.064					
CFA Model 2 Fit indices: CFI = 0.871, TLI = 0.855, RMSEA = 0.091, SRMR = 0.042					

The Structural Model

**Figure 2:**
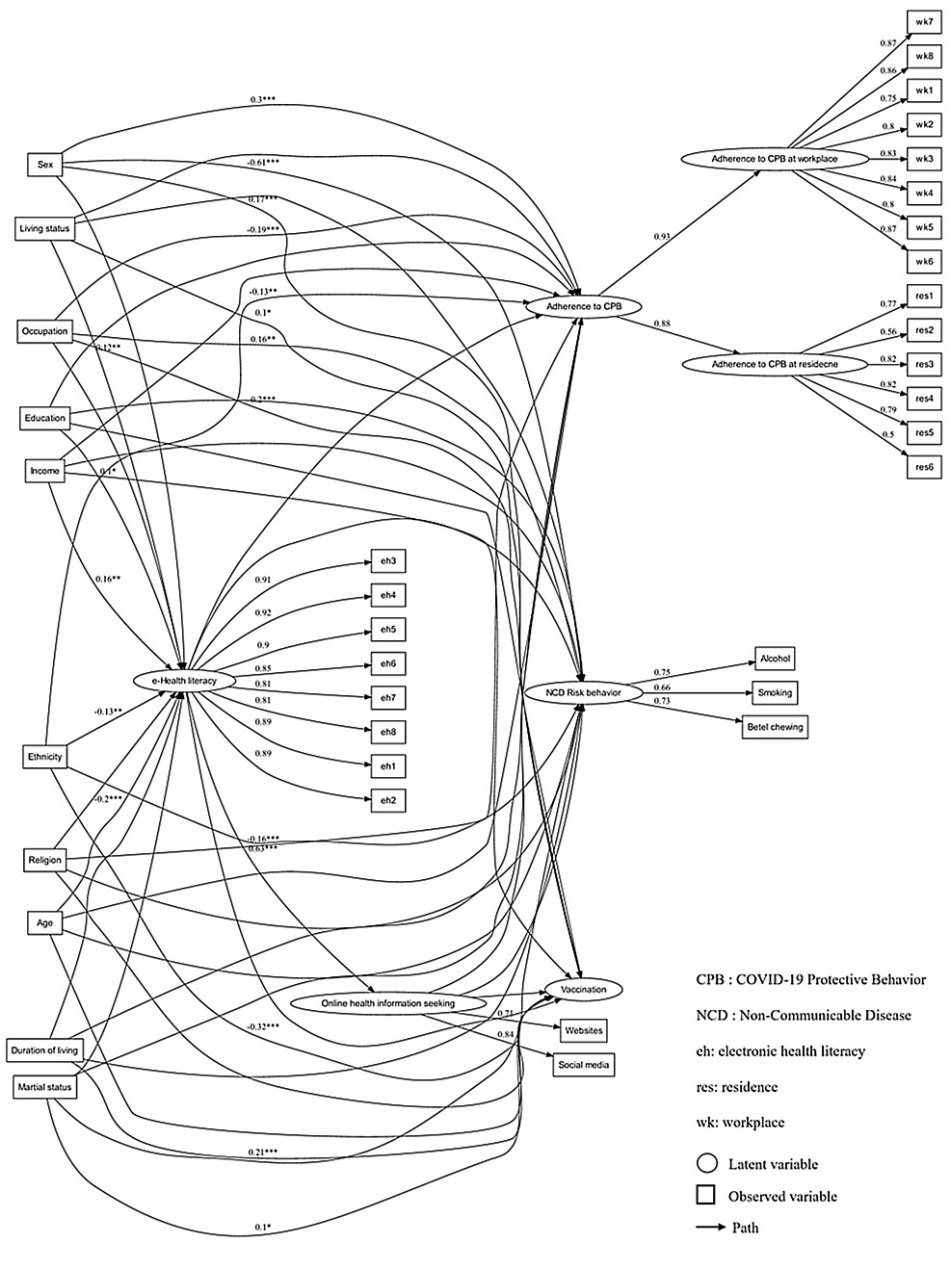
SEM showing standardized path coefficients of direct, indirect, and total effects of socio-demographic factors on COVID-19- and NCD-related behaviors, mediated through e-Health literacy and online health information-seeking behavior (* P<0.05, ** P<0.01, *** P<0.001)

The results of the structural model (structural path analysis) are visualized in Figure 2 and summarized in Table 6, highlighting only the pathways with significant direct and total effects. The structural model indicated the best fit indices (χ2 = 500.179, df = 622, p = 1.000, CFI = 0.956, TLI = 0.971, RMSEA = 0.029, SRMR = 0.043), revealing it fit the data well. It was noted that none of the indirect effects in the pathway from socio-demographic factors → e-Health literacy → online health information seeking → COVID-19, and NCD-related behaviors were found to be statistically significant, indicating the absence of serial mediation in our analysis.

**Table 6:** Standardized path coefficients of direct, indirect, and total effects of socio-demographic factors on COVID-19-related and NCD risk behaviors, mediated through e-Health literacy and online health information-seeking behavior NCD: non-communicable disease, CPB: COVID-19 protective behavior, CFI: comparative fit index, TLI: Tucker-Lewis index, RMSEA: root mean square error of approximation, SRMR: standardized root mean square residual, ^†^Referent group, NS: not significant * P < 0.05, ** P < 0.01, *** P < 0.001

Socio-demographic characteristics	NCD risk behavior			Adherence to CPB			COVID-19 vaccination		
	Direct effect	Indirect effect	Total effect	Direct effect	Indirect effect	Total effect	Direct effect	Indirect effect	Total effect
Sex (male^†^ vs. female)	-0.606***	-0.002	-0.614***	0.300***	0.002	0.307***	0.172***	-0.001	0.170***
Occupation (construction^† ^vs. factory vs. seafarers)	0.156**	0.001	0.160**	-0.190***	-0.001	-0.194***	0.204***	0.001	0.205***
Religion (Buddhist^†^ vs. non-Buddhist)	NS	NS	NS	-0.162**	0.000	-0.162**	NS	NS	NS
Ethnicity (Burmese^†^, non-Burmese)	NS	NS	NS	-0.135**	-0.005	-0.154**	-0.316***	0.003	-0.312***
Living status (alone^† ^vs. with family and relatives vs. with friends)	NS	NS	NS	NS	NS	NS	0.096*	-0.003	0.092*
Duration of stay (<5 years^† ^vs. 5 years and above)	NS	NS	NS	NS	NS	NS	0.208***	-0.002	0.205***
Model fit indices: CFI = 0.956, TLI = 0.971, RMSEA = 0.029, SRMR = 0.043									

Only significant direct and total effects of socio-demographic factors on the outcome behaviors were observed, with standardized path coefficients ranging from -0.606 to 0.300. Notably, these direct effects accounted for over three-fourths of the total effects. Regarding NCD risk behavior, gender showed a strong direct negative effect (β = -0.606), with females exhibiting reduced NCD risk behavior in comparison to males. Conversely, occupation had a moderately direct positive effect (β = 0.156), suggesting that seafarers showed higher NCD risk behavior than factory and construction workers. In terms of adherence to CPB, we observed a moderate direct positive effect of gender (β = 0.300), showing that females displayed higher adherence than males; a weak direct negative effect of occupation (β = -0.190), suggesting that seafarers exhibited lower adherence compared to factory and construction workers; a weak direct negative effect of religion (β = -0.162), revealing that non-Buddhist migrants showed lower adherence than Buddhists; and a weak direct negative effect of ethnicity (β = -0.135), indicating that non-Burmese migrants exhibited lower adherence than their Burmese counterparts. With regard to COVID-19 vaccination, we found a weak positive direct effect of gender (β = 0.172), indicating that females exhibited higher vaccination rates than males; a weak direct positive effect of occupation (β = 0.204), showing that seafarers displayed higher vaccination rates than factory and construction workers; a moderate negative direct effect of ethnicity (β = -0.316), revealing that non-Burmese migrants displayed lower vaccination than their Burmese counterparts; a weak positive direct effect of living status (β = 0.096), suggesting that migrants living with family, relatives, or friends had higher vaccination than those living alone; and a weak positive direct effect of duration of stay (β = 0.208), indicating that migrants living in Thailand for five years and above displayed higher vaccination than their counterparts.

## Discussion

This study represents the first serial mediation analysis examining the impact of e-Health literacy and online health information seeking on the association between socio-demographic factors and COVID-19- and NCD-related behaviors among vulnerable Myanmar migrants in Southern Thailand. The serial mediation analysis revealed that socio-demographic factors directly influenced COVID-19- and NCD-related behaviors, with no significant serial mediation roles observed for e-Health literacy and online health information seeking.

Although e-Health literacy [14] and online health information seeking [33] had mediating effects on health-related behaviors in recent literature in 2020 and 2022, our study did not find their mediating roles on COVID-19- and NCD-related behaviors. There are many explanations for this. In Thailand, the nationwide pandemic COVID-19 vaccination program was rolled out in 2021, and migrant populations were freely vaccinated [34], without consideration of their e-Health literacy status and online health information seeking. The Royal Thai government granted free access to COVID-19 vaccines for migrants and their family members [34], regardless of their legal status [16], potentially reducing migrants’ barriers to vaccine accessibility. There have been previous COVID-19 outbreaks among Myanmar migrant workers, as seen in factories and seafood markets in Samut Sakhon province during the 2020 COVID-19 second wave in Thailand [35]. This could potentially lead to higher vaccination rates among our study population, as migrants might be motivated to get vaccinated and employers might provide COVID-19 vaccine mandates for their migrant employees.

On the other hand, COVID-19 preventive measures were widely educated and implemented at their workplaces and residences via posters or talks [36]. Our study participants adhered to the recommended CPB, but not perfectly, as they exhibited a mean adherence score (±SD) of 6.7 out of 9 (± 2.0) at residences and 4.6 out of 8 (± 3.1) at workplaces. Other studies conducted in 2022 and 2023 also reported a high potential for COVID-19 prevention and control among Myanmar migrants in Thailand [37,38], elucidating that migrant workers exhibited a reasonably good understanding of COVID-19 and its preventive behaviors. As a result, they actively avoided risky behaviors and engaged in preventive measures [38].

It is also possible that during the COVID-19 pandemic, in conjunction with social distancing measures and limited availability [39], migrant populations may have altered or discontinued NCD risk behaviors [40]. Another explanation could be due to the decreasing prevalence of NCD risk behaviors, particularly in current tobacco use, in Thailand and Myanmar, as shown in the WHO Global Report on Trends in Prevalence of Tobacco Use 2000-2025 [41].

One of the key findings of our study is that the direct effects of socio-demographic factors were the main explanation for their associations with COVID-19- and NCD-related behaviors. One such factor is gender, which plays a significant role, with female migrants exhibiting lower NCD risk behaviors than males in our SEM analysis. A previous migrant study in Thailand in 2013 [42], as well as the 2009 and 2014 National-level WHO STEP Surveys [43] and the 2015-2016 Demographic Health Survey [44] in Myanmar, also reported higher rates of tobacco use, betel quid chewing, and alcohol consumption among males. In Myanmar culture and tradition, females face greater disapproval and stigma related to smoking and drinking, as these behaviors are seen as uncontrollable and wild behaviors of males [45]. In our study, females were more likely to adhere to CPB than males, which is consistent with a previous migrant study in 2020-2021 [46]. Compared to men, women may report a higher perceived risk and severity of COVID-19 infection [47], adopting more preventive practices. Our findings revealed that female migrants showed a higher COVID-19 vaccination uptake than males. Gender demonstrates controversial impacts on COVID-19 vaccine uptake, with no prediction in Australian refugees in 2021 [48], while it predicted uptake in Lebanese refugees in 2021 [49] and undocumented migrants in the US in 2020-2021 [50]. More research will be necessary to clarify the role of gender in COVID-19 vaccine uptake among these vulnerable populations.

Among our participants, seafarer migrants displayed high NCD risk behaviors, low adherence to CPB, and low COVID-19 vaccine uptake. Migrant seafarers have high rates of smoking and alcohol consumption among migrant seafarers [51,52] due to stressful working and living conditions [53], as well as high rates of COVID-19 infections due to close contact, the impracticality of wearing masks at sea, and small shared bedrooms [54]. These seafarers worked and stayed longer on the boats; it was impossible to practice physical distancing in the crowded working and living conditions [55], which may hamper their adherence to CPB. They could also face a lack of access to COVID-19 vaccines due to very long periods of isolation at sea. However, studies related to COVID-19 vaccine uptake among fishing communities in Thailand remain limited.

We found that non-Burmese migrants were associated with low COVID-19 vaccination uptake. Despite there being no similar study reporting the relationship between the different ethnic backgrounds of Myanmar migrants and COVID-19 vaccination uptake, a few studies reported varying outcomes among these demographic groups in Thailand. For instance, a study involving Myanmar seafarers in Pattani in 2016-2017 found that non-Burmese migrants were more prone to physical illnesses than Burmese migrants [51]. However, another study conducted in 2016-2017 revealed that non-Burmese ethnic (Dawei) seafarer families demonstrated better adaptation and coping mechanisms, with their children exhibiting lower malnutrition rates compared to other ethnic groups [56]. Further investigations into the diverse ethnic backgrounds of Myanmar migrants and their health-related behaviors should be explored more.

There were some limitations in our study. As our study was conducted in late 2022, there is a chance that people may have changed their COVID-19 preventive behaviors, impacting COVID-19-related outcomes. On reporting mediators and outcome measurements, it is challenging to prevent social desirability biases, but we mitigated this by using anonymous self-administered questionnaires and obtaining verbal consent only. Despite these limitations, our study is one of the limited studies that investigated both COVID-19- and NCD-related behaviors among vulnerable Myanmar migrants in Southern Thailand.

## Conclusions

Myanmar migrant workers in Southern Thailand had reasonably good practices in COVID-19-related behaviors, including high vaccination rates and good adherence to CPB. Additionally, these migrants are engaging in risky NCD-related behaviors, such as smoking, alcohol drinking, and betel chewing. The vast majority had a limited e-Health literacy level and did not search for online health information. Their COVID-19- and NCD-related behaviors were directly influenced by their socio-demographic factors, such as sex, occupation, ethnicity, religion, living status, and duration of living in Thailand. SEM analysis found no serial mediation effects of e-Health literacy and online health information seeking on the associations between socio-demographic factors and COVID-19-related as well as NCD-related behaviors. These findings highlighted that e-Health strategies may not be a high priority for enhancing migrants' health behaviors. Diverse interventions beyond e-Health strategies for future pandemic mitigation and improving health behaviors among vulnerable migrant populations are needed.

## References

[1] (2023). World Report on the Health of Refugees and Migrants. https://apps.who.int/iris/rest/bitstreams/1451966/retrieve.

[2] (2021). Thailand Migration Report 2019. https://thailand.un.org/en/50831-thailand-migration-report-2019.

[3] (2021). Impact of COVID-19 on Migrants in Thailand Situation Report. https://thailand.iom.int/sites/thailand/files/COVID19Response/UNMNW%20COVID19%20SitRep_September.pdf.

[4] (2021). COVID-19: impact on migrant workers and country response in Thailand. http://www.ilo.org/asia/publications/issue-briefs/WCMS_741920/lang--en/index.htm.

[5] Khai TS (2023). Socio-ecological barriers to access COVID-19 vaccination among Burmese irregular migrant workers in Thailand. J Migr Health.

[6] (2023). Health of Refugees and Migrants. https://cdn.who.int/media/docs/default-source/documents/publications/health-of-refugees-and-migrants-searo-20182ec00175-afa4-491e-8b18-eec0d418ff42.pdf.

[7] (2023). Prevention and Control of Noncommunicable Diseases in Thailand: the case for investment. https://thailand.un.org/sites/default/files/2021-11/%E6%9C%80%E6%96%B0%EF%BC%BFTHAILAND_NCD%20IC%20REPORT_v06_231121.pdf.

[8] Aung TN, Shirayama Y, Moolphate S, Lorga T, Yuasa M, Nyein Aung M (2020). Acculturation and its effects on health risk behaviors among Myanmar migrant workers: a cross-sectional survey in Chiang Mai, northern Thailand. Int J Environ Res Public Health.

[9] Kosiyaporn H, Julchoo S, Sinam P, Phaiyarom M, Kunpeuk W, Pudpong N, Suphanchaimat R (2020). Health literacy and its related determinants in migrant health workers and migrant health volunteers: a case study of Thailand, 2019. Int J Environ Res Public Health.

[10] Norman CD, Skinner HA (2006). eHealth literacy: essential skills for consumer health in a networked world. J Med Internet Res.

[11] Ameri F, Dastani M, Sabahi A (2022). The role of E-health literacy in preventive behaviors for COVID-19: a systematic review. J Health Lit.

[12] Wu Y, Wen J, Wang X (2022). Associations between e-health literacy and chronic disease self-management in older Chinese patients with chronic non-communicable diseases: a mediation analysis. BMC Public Health.

[13] Chen SC, Hong Nguyen NT, Lin CY (2023). Digital health literacy and well-being among university students: mediating roles of fear of COVID-19, information satisfaction, and internet information search. Digit Health.

[14] Bao X, Chen D, Shi L, Xia Y, Shi Z, Wang D (2022). The relationship between COVID-19-related prevention cognition and healthy lifestyle behaviors among university students: mediated by e-health literacy and self-efficacy. J Affect Disord.

[15] (2021). Office of Foreign Workers Administration: statistic. https://www.doe.go.th/prd/alien/statistic/param/site/152/cat/82/sub/73/pull/sub_category/view/list-label.

[16] (2023). Thailand’s Migrant Vaccination Programme Ensures No One Is Left Behind. https://www.who.int/thailand/news/detail/23-03-2022-thailand-s-migrant-vaccination-programme-ensures-no-one-is-left-behind.

[17] (2022). Keeping Safe From COVID-19: keeping safe in the new normal. https://www.who.int/southeastasia/outbreaks-and-emergencies/covid-19/What-can-we-do-to-keep-safe/protective-measures/workplace-precautions.

[18] (2021). Considerations for implementing and adjusting public health and social measures in the context of COVID-19. https://www.who.int/publications/i/item/considerations-in-adjusting-public-health-and-social-measures-in-the-context-of-covid-19-interim-guidance.

[19] (2021). Preventing and Mitigating COVID-19 at Work: policy brief, 19 May 2021. https://www.who.int/publications-detail-redirect/WHO-2019-nCoV-workplace-actions-policy-brief-2021-1.

[20] (2021). Community, Work, and School. https://www.cdc.gov/coronavirus/2019-ncov/community/index.html.

[21] (2022). Recommendations for Working Spaces and Organizations. https://ddc.moph.go.th/viralpneumonia/eng/file/recommendation/012working_space.pdf.

[22] (2022). Recommendation for Self-Prevention. https://ddc.moph.go.th/viralpneumonia/eng/info.php.

[23] Norman CD, Skinner HA (2006). eHEALS: the eHealth literacy scale. J Med Internet Res.

[24] (2021). Survey Tool and Guidance: rapid, simple, flexible behavioural insights on COVID-19: 29 July 2020. https://apps.who.int/iris/handle/10665/333549.

[25] Agarwal A, Ranjan P, Saikaustubh Y (2021). Development and validation of a questionnaire for assessing preventive practices and barriers among health care workers in COVID-19 pandemic. Indian J Med Microbiol.

[26] Tabachnick BG, Fidell LS (2021). Using multivariate statistics, 7th edition. Using Multivariate Statistics. Pearson Education.

[27] Watkins M (2021). A step-by-step guide to exploratory factor analysis with R and RStudio. https://www.routledge.com/A-Step-by-Step-Guide-to-Exploratory-Factor-Analysis-with-R-and-RStudio/Watkins/p/book/9780367634681#.

[28] Tavakol M, Wetzel A (2020). Factor analysis: a means for theory and instrument development in support of construct validity. Int J Med Educ.

[29] Lee S (2021). Exploratory factor analysis for a nursing workaround instrument in Korean and interpretations of statistical decision points. Comput Inform Nurs.

[30] Hu L, Bentler PM (1999). Cutoff criteria for fit indexes in covariance structure analysis: conventional criteria versus new alternatives. Struct Equ Modeling.

[31] de Sousa ÁFL, Teixeira JR, Lua I (2021). Determinants of COVID-19 vaccine hesitancy in Portuguese-speaking countries: a structural equations modeling approach. Vaccines (Basel).

[32] Yong AG, Pearce S (2013). A beginner’s guide to factor analysis: focusing on exploratory factor analysis. Tutor Quant Methods Psychol.

[33] Chen W, Zheng Q, Liang C, Xie Y, Gu D (2020). Factors influencing college students’ mental health promotion: the mediating effect of online mental health information seeking. Int J Environ Res Public Health.

[34] Tangcharoensathien V, Sachdev S, Viriyathorn S, Sriprasert K, Kongkam L, Srichomphu K, Patcharanarumol W (2022). Universal access to comprehensive COVID-19 services for everyone in Thailand. BMJ Glob Health.

[35] Rajatanavin N, Tuangratananon T, Suphanchaimat R, Tangcharoensathien V (2021). Responding to the COVID-19 second wave in Thailand by diversifying and adapting lessons from the first wave. BMJ Glob Health.

[36] (2023). Migrant Health Volunteers Trained to Help the Most Vulnerable During COVID-19. https://www.unicef.org/thailand/stories/migrant-health-volunteers-trained-help-most-vulnerable-during-covid-19.

[37] Hnuploy K, Sornlorm K, Soe TK (2022). COVID-19 vaccine acceptance and its determinants among Myanmar migrant workers in southern Thailand. Int J Environ Res Public Health.

[38] Noosorn N, Wongwat R (2023). The guideline development for COVID-19 control and prevention among Burmese migrant workers community at the Thai-Myanmar border. Health Sci J Thai.

[39] Panagiotidis P, Rantis K, Holeva V, Parlapani E, Diakogiannis I (2020). Changes in alcohol use habits in the general population, during the COVID-19 lockdown in Greece. Alcohol Alcohol.

[40] Klemperer EM, West JC, Peasley-Miklus C, Villanti AC (2020). Change in tobacco and electronic cigarette use and motivation to quit in response to COVID-19. Nicotine Tob Res.

[41] (2023). Who Global Report on Trends in Prevalence of Tobacco Use 2000-2025, Fourth Edition. https://www.who.int/publications-detail-redirect/9789240039322.

[42] Htin K, Howteerakull N, Suwannapong N, TipayamongkholgulI M (2014). Smoking, alcohol consumption and betal-quid chewing among young adult Myanmar laborers in Thailand. Southeast Asian J Trop Med Public Health.

[43] Grover S, Sinha DN, Gupta S, Gupta PC, Mehrotra R (2019). The changing face of risk factors for non-communicable disease in Myanmar: findings from the 2009 and 2014 WHO STEP Surveys. J Public Health (Oxf).

[44] Sreeramareddy CT, Aye SN, Venkateswaran SP (2021). Tobacco use and betel quid chewing among adults in Myanmar- estimates and social determinants from demographic and health survey, 2015-16. BMC Public Health.

[45] Howteerakul N, Suwannapong N, Than M (2005). Cigarette, alcohol use and physical activity among Myanmar youth workers, Samut Sakhon Province, Thailand. Southeast Asian J Trop Med Public Health.

[46] Skogberg N, Prinkey T, Lilja E, Koponen P, Castaneda AE (2023). Association of sociodemographic characteristics with self-perceived access to COVID-19 information and adherence to preventive measures among migrant origin and general populations in Finland: a cross-sectional study. BMJ Open.

[47] Alsharawy A, Spoon R, Smith A, Ball S (2023). Gender differences in fear and risk perception during the COVID-19 pandemic. Front Psychol.

[48] Liddell BJ, Murphy S, Mau V, Bryant R, O'Donnell M, McMahon T, Nickerson A (2021). Factors associated with COVID-19 vaccine hesitancy amongst refugees in Australia. Eur J Psychotraumatol.

[49] Salibi N, Abdulrahim S, El Haddad M, Bassil S, El Khoury Z, Ghattas H, McCall SJ (2021). COVID-19 vaccine acceptance in older Syrian refugees: Preliminary findings from an ongoing study. Prev Med Rep.

[50] Sudhinaraset M, Nwankwo E, Choi HY (2022). Immigration enforcement exposures and COVID-19 vaccine intentions among undocumented immigrants in California. Prev Med Rep.

[51] Kyaw PP, Geater AF (2021). Healthcare seeking preferences of Myanmar migrant seafarers in the deep south of Thailand. Int Marit Health.

[52] Annadurai K, Balan N, Ranaganathan K (2018). Non-communicable disease risk factor profile among Fishermen community of Kancheepuram district, Tamil Nadu: a cross sectional study. Int J Community Med Public Health.

[53] Warakit PK, Chaiwong S (2018). The quality of life of the migrant’s workers in ASEAN. Asia Pac J Rel Cul.

[54] Haritavorn N (2023). ‘Boat quarantine’: Lessons learned from SARS-CoV-2 prevention and control measures in fishing communities in Thailand. Int J Environ Res Public Health.

[55] (2023). Home Truths: access to adequate housing for migrant workers in the ASEAN region. https://www.ilo.org/wcmsp5/groups/public/---asia/---ro-bangkok/documents/publication/wcms_838972.pdf.

[56] Lwin SW, Geater AF (2019). Ethnic groups and father’s job influencing nutritional status of children (0-30 months) from Myanmar migrant community in southern Thailand. J Racial Ethn Health Disparities.

